# Supplementary figures and images for: Obstacle negotiation in female desert locust oviposition digging
Source: Naturwissenschaften. 2024 Aug 8;111(5):43. doi: 10.1007/s00114-024-01929-1 (PMC11310224; doi:10.1007/s00114-024-01929-1)

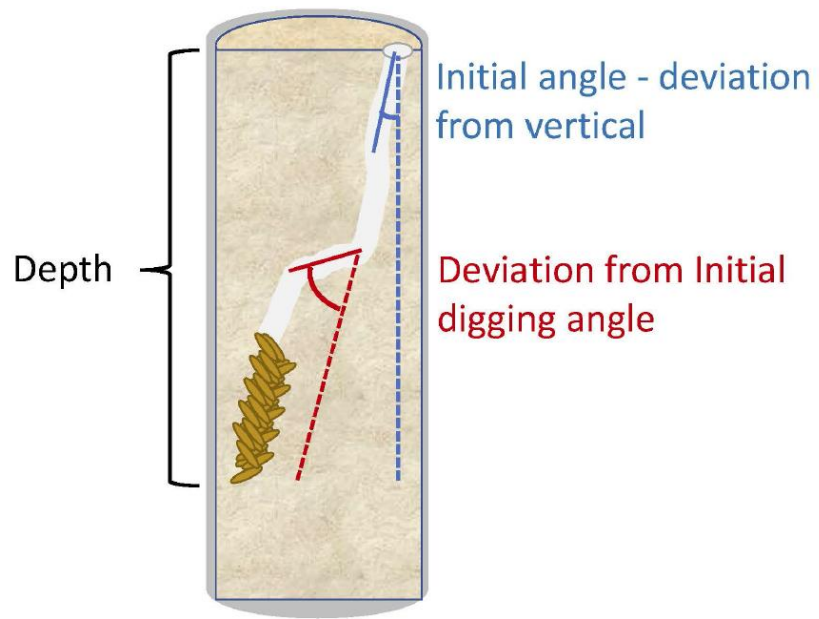

Figure S1

Supplement: Supplementary file 1 — Supplementary Figure S1 A schematic illustration of an oviposition tube and digging path depicting parameters of the digging path geometry that were quantified in this study, including the initial angle of the digging path (deviation from vertical), changes in the digging direction and deviation from the initial digging angle, and the depth reached (distance from the surface) (PDF 233 KB) [file 114_2024_1929_MOESM1_ESM.pdf]
